# 4-Methyl­phenyl quinoline-2-carboxyl­ate

**DOI:** 10.1107/S1600536812044030

**Published:** 2012-10-27

**Authors:** E. Fazal, Jerry P. Jasinski, Shannon T. Krauss, B. S. Sudha, H. S. Yathirajan

**Affiliations:** a Department of Chemistry, Yuvaraja’s College, Mysore 570 005, India; b Department of Chemistry, Keene State College, 229 Main Street, Keene, NH 03435-2001, USA; c Department of Studies in Chemistry, University of Mysore, Manasagangotri, Mysore 570 006, India

## Abstract

In the title compound, C_17_H_13_NO_2_, two mol­ecules crystallize in the asymmetric unit. The dihedral angle between the mean planes of the quinoline and benzene rings are 78.3 (4) and 88.2 (3)°. The carboxyl­ate group is twisted slightly from the quinoline ring by 7.1 (2) and 13.3 (4)°, respectively. In the crystal, weak C—H⋯O inter­actions are observed. Further stabilization is provided by weak π–π stacking inter­actions, with centroid–centroid distances of 3.564 (9)/3.689 (2) and 3.830 (1)/3.896 (5)Å, respectively.

## Related literature

For heterocycles in natural products, see: Morimoto *et al.* (1991 ▶); Michael (1997 ▶). For heterocycles in fragrances and dyes, see: Padwa *et al.* (1999 ▶). For heterocycles in biologically active compounds, see: Markees *et al.* (1970 ▶); Campbell *et al.* (1988 ▶). For quinoline alkaloids used as efficient drugs for the treatment of malaria, see: Robert & Meunier, (1998 ▶). For quinoline as a privileged scaffold in cancer drug discovery, see: Solomon & Lee (2011 ▶). For related structures, see: Dobrzyńska & Jerzykiewicz, (2004 ▶); Butcher *et al.* (2007 ▶); Jing & Qin (2008 ▶); Jasinski *et al.* (2010 ▶). For bond lengths, see Allen *et al.* (1987 ▶).
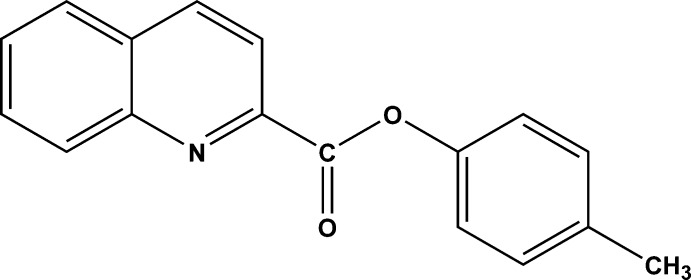


## Experimental

### 

#### Crystal data

C_17_H_13_NO_2_
*M* *_r_* = 263.28
Orthorhombic, 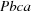

*a* = 11.5421 (2) Å
*b* = 17.3191 (3) Å
*c* = 26.6667 (5) Å
*V* = 5330.65 (16) Å^3^
*Z* = 16
Cu *K*α radiation
μ = 0.70 mm^−1^
*T* = 173 K
0.22 × 0.14 × 0.12 mm

#### Data collection

Oxford Diffraction Xcalibur (Eos, Gemini) diffractometer
Absorption correction: multi-scan (*CrysAlis RED*; Oxford Diffraction, 2010 ▶) *T*_min_ = 0.726, *T*_max_ = 1.000
34626 measured reflections
5265 independent reflections
4303 reflections with *I* > 2σ(*I*)
*R*_int_ = 0.046

#### Refinement

*R*[*F* ^2^ > 2σ(*F* ^2^)] = 0.044
*wR*(*F* ^2^) = 0.124
*S* = 1.02
5265 reflections
363 parameters
H-atom parameters constrained
Δρ_max_ = 0.20 e Å^−3^
Δρ_min_ = −0.19 e Å^−3^

### 

Data collection: *CrysAlis PRO* (Oxford Diffraction, 2010 ▶); cell refinement: *CrysAlis PRO*; data reduction: *CrysAlis RED* (Oxford Diffraction, 2010 ▶); program(s) used to solve structure: *SHELXS97* (Sheldrick, 2008 ▶); program(s) used to refine structure: *SHELXL97* (Sheldrick, 2008 ▶); molecular graphics: *SHELXTL* (Bruker, 2000 ▶); software used to prepare material for publication: *SHELXTL*.

## Supplementary Material

Crystal structure: contains datablock(s) global, I. DOI: 10.1107/S1600536812044030/bq2377sup1.cif

Structure factors: contains datablock(s) I. DOI: 10.1107/S1600536812044030/bq2377Isup2.hkl

Supplementary material file. DOI: 10.1107/S1600536812044030/bq2377Isup3.cml

Additional supplementary materials:  crystallographic information; 3D view; checkCIF report

## Figures and Tables

**Table 1:** Hydrogen-bond geometry (Å, °)

*D*—H⋯*A*	*D*—H	H⋯*A*	*D*⋯*A*	*D*—H⋯*A*
C15*B*—H15*B*⋯O2*A* ^i^	0.93	2.59	3.343 (2)	138

Symmetry code: (i) 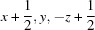.

**Fig. 1.**
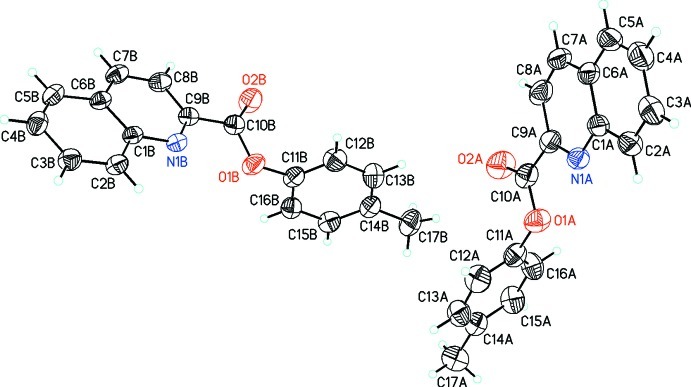
Molecular structure of the title compound showing the atom labeling scheme of two molecules (A & B) in the asymmetric unit and 50% probability displacement ellipsoids.

**Fig. 2.**
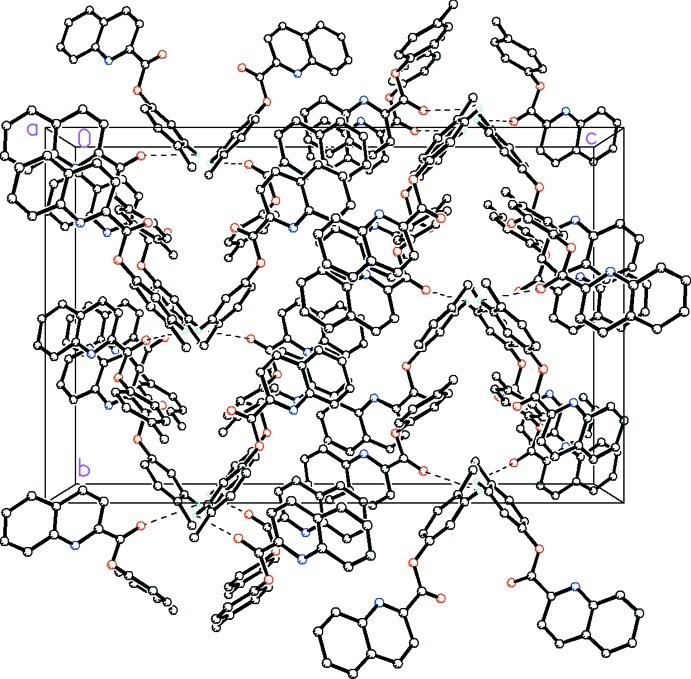
Packing diagram of the title compound viewed along the a axis. Dashed lines indicate weak C—H···O intermolecular interactions. The remaining H atoms have been removed for clarity.

## supplementary crystallographic information

### Comment

Quinoline-2-carboxylic acid derivatives are a class of important materials as anti-tuberculosis agents, as fluorescent reagents, hydrophobic field-detection reagents, visualization reagents, fluorescent labeled peptide probes and as antihyperglycemics. Quinoline derivatives represent a major class of heterocycles and are found in natural products (Morimoto *et al.*, 1991; Michael, 1997), numerous commercial products, including fragrances, dyes (Padwa *et al.*, 1999) and biologically active compounds (Markees *et al.*, 1970; Campbell *et al.*, 1988). Quinoline alkaloids such as quinine, chloroquin, mefloquine and amodiaquine are used as efficient drugs for the treatment of malaria (Robert & Meunier, 1998). Quinoline as a privileged scaffold in cancer drug discovery is published (Solomon & Lee, 2011). The crystal structures of quinoline-2-carboxylic acid (Dobrzyńska & Jerzykiewicz, 2004), 1-(quinolin-2-yl)ethanone (Butcher *et al.*, 2007) and methyl quinoline-2-carboxylate (Jing & Qin, 2008) and the synthesis, crystal structures and theoretical studies of four Schiff bases derived from 4-hydrazinyl-8-(trifluoromethyl) quinoline (Jasinski *et al.*, 2010) have been reported. In view of the importance of quinolines, the paper reports the crystal structure of the title compound, 4-methylphenyl quinoline-2-carboxylate, (I).

In the title compound, C_17_H_13_NO_2_, two molecules (A & B) crystallize in the asymmetric unit (Fig. 1). The dihedral angle between the mean planes of the quinoline and benzene rings are 78.3 (4)° (A) and 88.2 (3)° (B). The carboxylate group is twisted slightly from the quinoline ring by 7.1 (2)° (A) and 13.3 (4)° B, respectively. Bond lengths are in normal ranges (Allen *et al.*, (1987). In the crystal weak C—H···O intermolecular interactions are observed (Fig. 2). Further stabilization is provided by weak π–π stacking interactions with centroid to centroid distances of 3.564 (9)Å (Cg2-Cg1], 3.689 (2)Å (Cg2-Cg6), 3.830 (1)Å (Cg1-Cg5) and 3.896 (5)Å (Cg1-Cg1) [where Cg1 = N1A/C1A/C6A/C7A/C8A/C9A; Cg2 = C1A–C6A; Cg5 = N1B/C1B/C6B/C7B/C8B/C9B; C6 = C1B–C6B].

### Experimental

To a mixture of (1.73 g, 10 mmole) of quinaldic acid and p-cresol (1.08 g, 10 mmole) in a round-bottomed flask fitted with a reflux condenser with a drying tube, 0.75 g (5 mmole) of phosphorous oxychloride was added. The mixture was heated with occasional swirling, and temperature maintained at 348–353 K. At the end of six hours, the reaction mixture was poured into a solution of 2 g of sodium bicarbonate in 25 mL of water. The precipitated ester was filtered and washed with water. The yield of crude, air dried p-tolyl quinoline-2-carboxylate was 1.75 to 1.85 g (65-70%). X-ray quality crystals were obtained by recrystallization from absolute ethanol (m.p.: 396-398 K).

### Refinement

All of the H atoms were placed in their calculated positions and then refined using the riding model with Atom—H lengths of 0.93Å (CH) or 0.96Å (CH_3_). Isotropic displacement parameters for these atoms were set to 1.19-1.21 (CH) or 1.50 (CH_3_) times *U*_eq_ of the parent atom.

### Crystal data

**Table:**

C_17_H_13_NO_2_	*F*(000) = 2208
*M**_r_* = 263.28	*D*_x_ = 1.312 Mg m^−^^3^
Orthorhombic, *P**b**c**a*	Cu *K*α radiation, λ = 1.54184 Å
Hall symbol: -P 2ac 2ab	Cell parameters from 10490 reflections
*a* = 11.5421 (2) Å	θ = 3.8–72.7°
*b* = 17.3191 (3) Å	µ = 0.70 mm^−^^1^
*c* = 26.6667 (5) Å	*T* = 173 K
*V* = 5330.65 (16) Å^3^	Chunk, colorless
*Z* = 16	0.22 × 0.14 × 0.12 mm

### Data collection

**Table:**

Oxford Diffraction Xcalibur (Eos, Gemini) diffractometer	5265 independent reflections
Radiation source: Enhance (Cu) X-ray Source	4303 reflections with *I* > 2σ(*I*)
Graphite monochromator	*R*_int_ = 0.046
Detector resolution: 16.0416 pixels mm^-1^	θ_max_ = 72.8°, θ_min_ = 4.9°
ω scans	*h* = −14→10
Absorption correction: multi-scan (*CrysAlis PRO* and *CrysAlis RED*; Oxford Diffraction, 2010)	*k* = −20→21
*T*_min_ = 0.726, *T*_max_ = 1.000	*l* = −32→31
34626 measured reflections	

### Refinement

**Table:**

Refinement on *F*^2^	Primary atom site location: structure-invariant direct methods
Least-squares matrix: full	Secondary atom site location: difference Fourier map
*R*[*F*^2^ > 2σ(*F*^2^)] = 0.044	Hydrogen site location: inferred from neighbouring sites
*wR*(*F*^2^) = 0.124	H-atom parameters constrained
*S* = 1.02	*w* = 1/[σ^2^(*F*_o_^2^) + (0.0604*P*)^2^ + 1.783*P*] where *P* = (*F*_o_^2^ + 2*F*_c_^2^)/3
5265 reflections	(Δ/σ)_max_ = 0.001
363 parameters	Δρ_max_ = 0.20 e Å^−^^3^
0 restraints	Δρ_min_ = −0.19 e Å^−^^3^

### Special details

**Table:**

Geometry. All esds (except the esd in the dihedral angle between two l.s. planes) are estimated using the full covariance matrix. The cell esds are taken into account individually in the estimation of esds in distances, angles and torsion angles; correlations between esds in cell parameters are only used when they are defined by crystal symmetry. An approximate (isotropic) treatment of cell esds is used for estimating esds involving l.s. planes.
Refinement. Refinement of *F*^2^ against ALL reflections. The weighted *R*-factor *wR* and goodness of fit *S* are based on *F*^2^, conventional *R*-factors *R* are based on *F*, with *F* set to zero for negative *F*^2^. The threshold expression of *F*^2^ > σ(*F*^2^) is used only for calculating *R*-factors(gt) *etc*. and is not relevant to the choice of reflections for refinement. *R*-factors based on *F*^2^ are statistically about twice as large as those based on *F*, and *R*- factors based on ALL data will be even larger.

### Fractional atomic coordinates and isotropic or equivalent isotropic displacement parameters (Å^2^)

**Table:**

	*x*	*y*	*z*	*U*_iso_*/*U*_eq_	
O1A	0.12216 (11)	0.67251 (7)	0.39057 (4)	0.0511 (3)	
O2A	0.13223 (13)	0.56857 (9)	0.34041 (5)	0.0657 (4)	
N1A	−0.03742 (11)	0.60902 (8)	0.44686 (5)	0.0390 (3)	
C1A	−0.11347 (13)	0.57558 (9)	0.47931 (6)	0.0396 (4)	
C2A	−0.14961 (14)	0.61816 (10)	0.52172 (6)	0.0450 (4)	
H2A	−0.1244	0.6688	0.5259	0.054*	
C3A	−0.22121 (15)	0.58568 (11)	0.55662 (7)	0.0525 (4)	
H3A	−0.2437	0.6142	0.5845	0.063*	
C4A	−0.26120 (16)	0.50963 (12)	0.55081 (8)	0.0578 (5)	
H4A	−0.3097	0.4880	0.5749	0.069*	
C5A	−0.22915 (15)	0.46757 (11)	0.51017 (8)	0.0569 (5)	
H5A	−0.2561	0.4172	0.5067	0.068*	
C6A	−0.15524 (14)	0.49894 (9)	0.47282 (7)	0.0464 (4)	
C7A	−0.11881 (16)	0.45862 (10)	0.43001 (8)	0.0537 (5)	
H7A	−0.1462	0.4090	0.4238	0.064*	
C8A	−0.04317 (17)	0.49246 (10)	0.39768 (7)	0.0526 (5)	
H8A	−0.0185	0.4664	0.3691	0.063*	
C9A	−0.00251 (14)	0.56800 (9)	0.40816 (6)	0.0422 (4)	
C10A	0.08924 (16)	0.60161 (10)	0.37521 (6)	0.0455 (4)	
C11A	0.21459 (15)	0.70870 (10)	0.36529 (6)	0.0447 (4)	
C12A	0.32213 (17)	0.70819 (12)	0.38707 (7)	0.0555 (5)	
H12A	0.3348	0.6815	0.4168	0.067*	
C13A	0.41123 (16)	0.74779 (11)	0.36423 (7)	0.0534 (4)	
H13A	0.4842	0.7475	0.3790	0.064*	
C14A	0.39551 (15)	0.78789 (10)	0.32009 (6)	0.0452 (4)	
C15A	0.28520 (17)	0.78769 (12)	0.29953 (7)	0.0553 (5)	
H15A	0.2719	0.8147	0.2699	0.066*	
C16A	0.19469 (16)	0.74867 (12)	0.32171 (7)	0.0535 (5)	
H16A	0.1212	0.7494	0.3074	0.064*	
C17A	0.49506 (18)	0.82914 (13)	0.29514 (7)	0.0600 (5)	
H17D	0.5132	0.8040	0.2640	0.090*	
H17E	0.5616	0.8278	0.3167	0.090*	
H17F	0.4738	0.8818	0.2888	0.090*	
O1B	0.66824 (11)	0.35457 (6)	0.36695 (4)	0.0466 (3)	
O2B	0.60648 (11)	0.25607 (7)	0.31907 (5)	0.0540 (3)	
N1B	0.78323 (10)	0.26045 (7)	0.42615 (4)	0.0322 (3)	
C1B	0.85486 (12)	0.21554 (8)	0.45442 (5)	0.0309 (3)	
C2B	0.90207 (13)	0.24698 (9)	0.49877 (6)	0.0365 (3)	
H2B	0.8820	0.2968	0.5085	0.044*	
C3B	0.97676 (14)	0.20498 (9)	0.52742 (6)	0.0396 (3)	
H3B	1.0079	0.2265	0.5564	0.048*	
C4B	1.00717 (14)	0.12907 (9)	0.51339 (6)	0.0409 (4)	
H4B	1.0576	0.1007	0.5334	0.049*	
C5B	0.96322 (14)	0.09699 (9)	0.47076 (6)	0.0392 (4)	
H5B	0.9842	0.0470	0.4618	0.047*	
C6B	0.88617 (13)	0.13903 (8)	0.44012 (5)	0.0330 (3)	
C7B	0.83864 (14)	0.11000 (9)	0.39521 (6)	0.0385 (3)	
H7B	0.8567	0.0603	0.3846	0.046*	
C8B	0.76635 (14)	0.15486 (9)	0.36753 (6)	0.0385 (3)	
H8B	0.7338	0.1363	0.3380	0.046*	
C9B	0.74165 (12)	0.23042 (8)	0.38463 (5)	0.0332 (3)	
C10B	0.66380 (13)	0.27980 (9)	0.35296 (5)	0.0362 (3)	
C11B	0.60426 (15)	0.40810 (9)	0.33826 (6)	0.0396 (4)	
C12B	0.48842 (16)	0.41864 (10)	0.34708 (6)	0.0481 (4)	
H12B	0.4496	0.3877	0.3701	0.058*	
C13B	0.42981 (16)	0.47621 (11)	0.32107 (6)	0.0488 (4)	
H13B	0.3511	0.4835	0.3269	0.059*	
C14B	0.48616 (15)	0.52276 (9)	0.28679 (6)	0.0428 (4)	
C15B	0.60316 (15)	0.51005 (10)	0.27840 (6)	0.0428 (4)	
H15B	0.6422	0.5403	0.2551	0.051*	
C16B	0.66314 (15)	0.45307 (9)	0.30413 (6)	0.0416 (4)	
H16B	0.7418	0.4454	0.2984	0.050*	
C17B	0.42202 (19)	0.58559 (11)	0.25908 (8)	0.0597 (5)	
H17A	0.4712	0.6300	0.2558	0.089*	
H17B	0.3536	0.5994	0.2775	0.089*	
H17C	0.4005	0.5673	0.2264	0.089*	

### Atomic displacement parameters (Å^2^)

**Table:**

	*U*^11^	*U*^22^	*U*^33^	*U*^12^	*U*^13^	*U*^23^
O1A	0.0630 (8)	0.0459 (7)	0.0443 (6)	−0.0036 (6)	0.0113 (6)	−0.0089 (5)
O2A	0.0815 (10)	0.0666 (9)	0.0489 (7)	0.0005 (8)	0.0093 (7)	−0.0219 (6)
N1A	0.0399 (7)	0.0362 (7)	0.0408 (7)	0.0000 (5)	−0.0069 (6)	−0.0042 (5)
C1A	0.0340 (8)	0.0357 (8)	0.0491 (9)	0.0000 (6)	−0.0108 (7)	0.0032 (7)
C2A	0.0400 (9)	0.0456 (9)	0.0493 (9)	−0.0030 (7)	−0.0047 (7)	−0.0009 (7)
C3A	0.0397 (9)	0.0626 (12)	0.0552 (10)	0.0000 (8)	−0.0009 (8)	0.0061 (9)
C4A	0.0396 (9)	0.0603 (12)	0.0735 (13)	0.0004 (8)	−0.0007 (9)	0.0205 (10)
C5A	0.0391 (9)	0.0409 (9)	0.0908 (15)	−0.0038 (7)	−0.0120 (9)	0.0193 (10)
C6A	0.0384 (8)	0.0339 (8)	0.0670 (11)	0.0023 (7)	−0.0151 (8)	0.0030 (8)
C7A	0.0485 (10)	0.0333 (8)	0.0793 (13)	−0.0010 (7)	−0.0175 (9)	−0.0060 (8)
C8A	0.0572 (11)	0.0427 (9)	0.0580 (11)	0.0080 (8)	−0.0130 (9)	−0.0156 (8)
C9A	0.0438 (9)	0.0388 (8)	0.0439 (8)	0.0053 (7)	−0.0119 (7)	−0.0067 (7)
C10A	0.0544 (10)	0.0463 (9)	0.0357 (8)	0.0079 (8)	−0.0076 (7)	−0.0088 (7)
C11A	0.0535 (10)	0.0437 (9)	0.0368 (8)	0.0051 (8)	0.0066 (7)	−0.0038 (7)
C12A	0.0650 (12)	0.0597 (11)	0.0418 (9)	0.0046 (9)	−0.0070 (9)	0.0130 (8)
C13A	0.0509 (10)	0.0619 (11)	0.0475 (10)	0.0043 (9)	−0.0101 (8)	0.0077 (8)
C14A	0.0526 (10)	0.0464 (9)	0.0366 (8)	0.0057 (8)	0.0002 (7)	−0.0019 (7)
C15A	0.0584 (11)	0.0684 (12)	0.0390 (9)	0.0069 (9)	−0.0039 (8)	0.0129 (8)
C16A	0.0452 (9)	0.0704 (12)	0.0447 (9)	0.0062 (9)	−0.0045 (8)	0.0059 (9)
C17A	0.0640 (12)	0.0682 (13)	0.0477 (10)	−0.0078 (10)	0.0010 (9)	0.0003 (9)
O1B	0.0612 (7)	0.0323 (6)	0.0463 (6)	0.0021 (5)	−0.0194 (5)	−0.0004 (5)
O2B	0.0662 (8)	0.0464 (7)	0.0494 (7)	0.0073 (6)	−0.0224 (6)	−0.0120 (5)
N1B	0.0362 (6)	0.0270 (6)	0.0333 (6)	−0.0016 (5)	0.0012 (5)	−0.0006 (5)
C1B	0.0337 (7)	0.0259 (7)	0.0330 (7)	−0.0026 (6)	0.0037 (6)	0.0012 (5)
C2B	0.0435 (8)	0.0283 (7)	0.0378 (8)	−0.0004 (6)	−0.0009 (6)	−0.0010 (6)
C3B	0.0460 (9)	0.0366 (8)	0.0363 (8)	−0.0016 (7)	−0.0036 (7)	0.0017 (6)
C4B	0.0433 (9)	0.0379 (8)	0.0417 (8)	0.0057 (7)	−0.0005 (7)	0.0083 (7)
C5B	0.0466 (9)	0.0270 (7)	0.0441 (8)	0.0049 (6)	0.0069 (7)	0.0036 (6)
C6B	0.0371 (8)	0.0267 (7)	0.0351 (7)	−0.0019 (6)	0.0083 (6)	0.0007 (5)
C7B	0.0488 (9)	0.0265 (7)	0.0400 (8)	0.0000 (6)	0.0061 (7)	−0.0051 (6)
C8B	0.0467 (9)	0.0343 (8)	0.0343 (7)	−0.0045 (7)	0.0007 (6)	−0.0068 (6)
C9B	0.0346 (7)	0.0318 (7)	0.0332 (7)	−0.0036 (6)	0.0020 (6)	−0.0009 (6)
C10B	0.0385 (8)	0.0367 (8)	0.0335 (7)	−0.0013 (6)	0.0014 (6)	−0.0026 (6)
C11B	0.0514 (9)	0.0332 (8)	0.0341 (8)	0.0026 (7)	−0.0108 (7)	−0.0023 (6)
C12B	0.0519 (10)	0.0484 (10)	0.0440 (9)	−0.0026 (8)	0.0000 (8)	0.0086 (7)
C13B	0.0458 (9)	0.0545 (10)	0.0462 (9)	0.0072 (8)	−0.0011 (8)	0.0022 (8)
C14B	0.0528 (10)	0.0387 (8)	0.0369 (8)	0.0061 (7)	−0.0073 (7)	−0.0029 (6)
C15B	0.0533 (10)	0.0414 (9)	0.0337 (8)	−0.0005 (7)	−0.0022 (7)	0.0030 (6)
C16B	0.0444 (9)	0.0426 (9)	0.0378 (8)	0.0034 (7)	−0.0022 (7)	−0.0044 (7)
C17B	0.0685 (12)	0.0532 (11)	0.0573 (11)	0.0158 (9)	−0.0089 (10)	0.0076 (9)

### Geometric parameters (Å, º)

**Table:**

O1A—C10A	1.349 (2)	O1B—C10B	1.3486 (18)
O1A—C11A	1.409 (2)	O1B—C11B	1.4108 (18)
O2A—C10A	1.198 (2)	O2B—C10B	1.1930 (18)
N1A—C9A	1.316 (2)	N1B—C9B	1.3139 (18)
N1A—C1A	1.362 (2)	N1B—C1B	1.3626 (18)
C1A—C2A	1.413 (2)	C1B—C2B	1.412 (2)
C1A—C6A	1.423 (2)	C1B—C6B	1.4254 (19)
C2A—C3A	1.366 (2)	C2B—C3B	1.362 (2)
C2A—H2A	0.9300	C2B—H2B	0.9300
C3A—C4A	1.404 (3)	C3B—C4B	1.411 (2)
C3A—H3A	0.9300	C3B—H3B	0.9300
C4A—C5A	1.357 (3)	C4B—C5B	1.363 (2)
C4A—H4A	0.9300	C4B—H4B	0.9300
C5A—C6A	1.419 (3)	C5B—C6B	1.410 (2)
C5A—H5A	0.9300	C5B—H5B	0.9300
C6A—C7A	1.403 (3)	C6B—C7B	1.410 (2)
C7A—C8A	1.360 (3)	C7B—C8B	1.358 (2)
C7A—H7A	0.9300	C7B—H7B	0.9300
C8A—C9A	1.418 (2)	C8B—C9B	1.415 (2)
C8A—H8A	0.9300	C8B—H8B	0.9300
C9A—C10A	1.494 (3)	C9B—C10B	1.501 (2)
C11A—C12A	1.370 (2)	C11B—C12B	1.370 (2)
C11A—C16A	1.372 (2)	C11B—C16B	1.377 (2)
C12A—C13A	1.378 (3)	C12B—C13B	1.390 (2)
C12A—H12A	0.9300	C12B—H12B	0.9300
C13A—C14A	1.379 (2)	C13B—C14B	1.382 (2)
C13A—H13A	0.9300	C13B—H13B	0.9300
C14A—C15A	1.386 (2)	C14B—C15B	1.386 (2)
C14A—C17A	1.508 (3)	C14B—C17B	1.509 (2)
C15A—C16A	1.378 (3)	C15B—C16B	1.387 (2)
C15A—H15A	0.9300	C15B—H15B	0.9300
C16A—H16A	0.9300	C16B—H16B	0.9300
C17A—H17D	0.9600	C17B—H17A	0.9600
C17A—H17E	0.9600	C17B—H17B	0.9600
C17A—H17F	0.9600	C17B—H17C	0.9600
			
C10A—O1A—C11A	118.21 (13)	C10B—O1B—C11B	117.46 (12)
C9A—N1A—C1A	117.78 (14)	C9B—N1B—C1B	117.50 (12)
N1A—C1A—C2A	118.48 (14)	N1B—C1B—C2B	118.52 (12)
N1A—C1A—C6A	122.53 (16)	N1B—C1B—C6B	122.45 (13)
C2A—C1A—C6A	118.96 (16)	C2B—C1B—C6B	119.01 (13)
C3A—C2A—C1A	120.60 (17)	C3B—C2B—C1B	120.54 (14)
C3A—C2A—H2A	119.7	C3B—C2B—H2B	119.7
C1A—C2A—H2A	119.7	C1B—C2B—H2B	119.7
C2A—C3A—C4A	120.66 (19)	C2B—C3B—C4B	120.41 (15)
C2A—C3A—H3A	119.7	C2B—C3B—H3B	119.8
C4A—C3A—H3A	119.7	C4B—C3B—H3B	119.8
C5A—C4A—C3A	120.14 (18)	C5B—C4B—C3B	120.54 (15)
C5A—C4A—H4A	119.9	C5B—C4B—H4B	119.7
C3A—C4A—H4A	119.9	C3B—C4B—H4B	119.7
C4A—C5A—C6A	121.24 (17)	C4B—C5B—C6B	120.49 (14)
C4A—C5A—H5A	119.4	C4B—C5B—H5B	119.8
C6A—C5A—H5A	119.4	C6B—C5B—H5B	119.8
C7A—C6A—C5A	124.10 (17)	C7B—C6B—C5B	123.60 (14)
C7A—C6A—C1A	117.51 (17)	C7B—C6B—C1B	117.39 (14)
C5A—C6A—C1A	118.38 (17)	C5B—C6B—C1B	119.00 (14)
C8A—C7A—C6A	119.59 (16)	C8B—C7B—C6B	119.78 (14)
C8A—C7A—H7A	120.2	C8B—C7B—H7B	120.1
C6A—C7A—H7A	120.2	C6B—C7B—H7B	120.1
C7A—C8A—C9A	119.04 (17)	C7B—C8B—C9B	118.53 (14)
C7A—C8A—H8A	120.5	C7B—C8B—H8B	120.7
C9A—C8A—H8A	120.5	C9B—C8B—H8B	120.7
N1A—C9A—C8A	123.46 (17)	N1B—C9B—C8B	124.34 (14)
N1A—C9A—C10A	117.90 (14)	N1B—C9B—C10B	117.87 (13)
C8A—C9A—C10A	118.56 (15)	C8B—C9B—C10B	117.79 (13)
O2A—C10A—O1A	123.60 (18)	O2B—C10B—O1B	124.15 (14)
O2A—C10A—C9A	124.28 (17)	O2B—C10B—C9B	124.21 (14)
O1A—C10A—C9A	112.06 (14)	O1B—C10B—C9B	111.63 (12)
C12A—C11A—C16A	120.93 (17)	C12B—C11B—C16B	121.32 (15)
C12A—C11A—O1A	118.69 (15)	C12B—C11B—O1B	120.35 (15)
C16A—C11A—O1A	120.19 (16)	C16B—C11B—O1B	118.14 (15)
C11A—C12A—C13A	119.03 (16)	C11B—C12B—C13B	119.00 (16)
C11A—C12A—H12A	120.5	C11B—C12B—H12B	120.5
C13A—C12A—H12A	120.5	C13B—C12B—H12B	120.5
C12A—C13A—C14A	122.00 (17)	C14B—C13B—C12B	121.30 (17)
C12A—C13A—H13A	119.0	C14B—C13B—H13B	119.4
C14A—C13A—H13A	119.0	C12B—C13B—H13B	119.4
C13A—C14A—C15A	117.20 (17)	C13B—C14B—C15B	118.22 (15)
C13A—C14A—C17A	121.00 (16)	C13B—C14B—C17B	120.91 (17)
C15A—C14A—C17A	121.79 (16)	C15B—C14B—C17B	120.87 (16)
C16A—C15A—C14A	121.88 (16)	C14B—C15B—C16B	121.28 (16)
C16A—C15A—H15A	119.1	C14B—C15B—H15B	119.4
C14A—C15A—H15A	119.1	C16B—C15B—H15B	119.4
C11A—C16A—C15A	118.95 (17)	C11B—C16B—C15B	118.88 (16)
C11A—C16A—H16A	120.5	C11B—C16B—H16B	120.6
C15A—C16A—H16A	120.5	C15B—C16B—H16B	120.6
C14A—C17A—H17D	109.5	C14B—C17B—H17A	109.5
C14A—C17A—H17E	109.5	C14B—C17B—H17B	109.5
H17D—C17A—H17E	109.5	H17A—C17B—H17B	109.5
C14A—C17A—H17F	109.5	C14B—C17B—H17C	109.5
H17D—C17A—H17F	109.5	H17A—C17B—H17C	109.5
H17E—C17A—H17F	109.5	H17B—C17B—H17C	109.5
			
C9A—N1A—C1A—C2A	178.47 (14)	C9B—N1B—C1B—C2B	179.00 (13)
C9A—N1A—C1A—C6A	0.2 (2)	C9B—N1B—C1B—C6B	0.8 (2)
N1A—C1A—C2A—C3A	−176.71 (15)	N1B—C1B—C2B—C3B	−178.08 (13)
C6A—C1A—C2A—C3A	1.6 (2)	C6B—C1B—C2B—C3B	0.2 (2)
C1A—C2A—C3A—C4A	−0.6 (3)	C1B—C2B—C3B—C4B	−0.6 (2)
C2A—C3A—C4A—C5A	−0.2 (3)	C2B—C3B—C4B—C5B	0.7 (2)
C3A—C4A—C5A—C6A	0.0 (3)	C3B—C4B—C5B—C6B	−0.3 (2)
C4A—C5A—C6A—C7A	179.93 (17)	C4B—C5B—C6B—C7B	179.09 (15)
C4A—C5A—C6A—C1A	1.1 (3)	C4B—C5B—C6B—C1B	−0.1 (2)
N1A—C1A—C6A—C7A	−2.5 (2)	N1B—C1B—C6B—C7B	−0.9 (2)
C2A—C1A—C6A—C7A	179.24 (15)	C2B—C1B—C6B—C7B	−179.09 (13)
N1A—C1A—C6A—C5A	176.45 (14)	N1B—C1B—C6B—C5B	178.34 (13)
C2A—C1A—C6A—C5A	−1.8 (2)	C2B—C1B—C6B—C5B	0.1 (2)
C5A—C6A—C7A—C8A	−176.68 (17)	C5B—C6B—C7B—C8B	−179.01 (14)
C1A—C6A—C7A—C8A	2.2 (2)	C1B—C6B—C7B—C8B	0.2 (2)
C6A—C7A—C8A—C9A	0.2 (3)	C6B—C7B—C8B—C9B	0.6 (2)
C1A—N1A—C9A—C8A	2.4 (2)	C1B—N1B—C9B—C8B	0.0 (2)
C1A—N1A—C9A—C10A	−174.34 (13)	C1B—N1B—C9B—C10B	−179.30 (12)
C7A—C8A—C9A—N1A	−2.7 (3)	C7B—C8B—C9B—N1B	−0.7 (2)
C7A—C8A—C9A—C10A	174.08 (16)	C7B—C8B—C9B—C10B	178.62 (14)
C11A—O1A—C10A—O2A	−2.1 (3)	C11B—O1B—C10B—O2B	−2.6 (2)
C11A—O1A—C10A—C9A	175.13 (14)	C11B—O1B—C10B—C9B	176.32 (13)
N1A—C9A—C10A—O2A	176.18 (17)	N1B—C9B—C10B—O2B	−167.71 (15)
C8A—C9A—C10A—O2A	−0.7 (3)	C8B—C9B—C10B—O2B	12.9 (2)
N1A—C9A—C10A—O1A	−1.0 (2)	N1B—C9B—C10B—O1B	13.42 (19)
C8A—C9A—C10A—O1A	−177.91 (15)	C8B—C9B—C10B—O1B	−165.96 (13)
C10A—O1A—C11A—C12A	−101.26 (19)	C10B—O1B—C11B—C12B	82.90 (19)
C10A—O1A—C11A—C16A	83.7 (2)	C10B—O1B—C11B—C16B	−101.91 (17)
C16A—C11A—C12A—C13A	−0.8 (3)	C16B—C11B—C12B—C13B	−0.3 (3)
O1A—C11A—C12A—C13A	−175.76 (16)	O1B—C11B—C12B—C13B	174.77 (15)
C11A—C12A—C13A—C14A	0.0 (3)	C11B—C12B—C13B—C14B	−0.1 (3)
C12A—C13A—C14A—C15A	0.7 (3)	C12B—C13B—C14B—C15B	0.8 (3)
C12A—C13A—C14A—C17A	−178.47 (18)	C12B—C13B—C14B—C17B	−179.43 (17)
C13A—C14A—C15A—C16A	−0.6 (3)	C13B—C14B—C15B—C16B	−1.0 (2)
C17A—C14A—C15A—C16A	178.60 (19)	C17B—C14B—C15B—C16B	179.21 (16)
C12A—C11A—C16A—C15A	0.9 (3)	C12B—C11B—C16B—C15B	0.1 (2)
O1A—C11A—C16A—C15A	175.81 (17)	O1B—C11B—C16B—C15B	−175.10 (13)
C14A—C15A—C16A—C11A	−0.2 (3)	C14B—C15B—C16B—C11B	0.6 (2)

### Hydrogen-bond geometry (Å, º)

**Table:**

*D*—H···*A*	*D*—H	H···*A*	*D*···*A*	*D*—H···*A*
C15*B*—H15*B*···O2*A*^i^	0.93	2.59	3.343 (2)	138

Symmetry code: (i) *x*+1/2, *y*, −*z*+1/2.
